# Psychological Antecedents of the Intention to Open the Windows at Home and Exposure to a Ventilation Recommendation

**DOI:** 10.3389/fpsyg.2022.872626

**Published:** 2022-05-13

**Authors:** François Durand, Barbara Bonnefoy, Dorothée Marchand, Thierry Meyer

**Affiliations:** ^1^Laboratoire Parisien de Psychologie Sociale, Paris Nanterre University, Nanterre, France; ^2^Centre Scientifique et Technique du Bâtiment, Champs-sur-Marne, France

**Keywords:** indoor air quality, odor awareness, habits, health recommendations, window-opening, theory of planned behavior (TPB), home

## Abstract

**Objective:**

The psychological antecedents of the intention to open the windows at home were explored through the Theory of Planned Behavior (TPB), supplemented with Habits regarding the behavior and contextual factors.

**Design:**

A four-treatment design compared the effect of an exposure to a recommendation about home ventilation and the effect of one’s own awareness odors (negative vs. positive) as a manipulated variable. Two quasi-experimental online surveys were conducted. A student sample (Study 1; *N* = 159) was replicated with a general population sample (Study 2; *N* = 338).

**Results:**

Multiple hierarchical regression models were conducted (3 for Study 1; 3 for Study 2). The extended TPB model provided stable predictors explaining around sixty percent of variance. Attitude and Habits were the main predictors of window openings, Perceived Behavioral control as a secondary predictor. Perceived Behavioral Control contributed significatively with a negative interaction with Attitudes. Odor awareness decreased Intention to manually ventilate. No effect of recommendation was observed.

**Discussion:**

The results filled a gap in the literature about the willingness to manually ventilate at home and efficacy of a recommendation. Practical implications argue that beyond a generic recommendation, effective messages need to be tailored regarding the determinants of willingness to open the windows.

## Introduction

Around the world, the majority of people spend most of their time indoors (80–90%) (González-Martín et al., 2020). A secure and comfortable home or apartment is far from a healthy environment, with sources of household air pollution (Hoskins, 2010; Pérez-Padilla et al., 2010; Apte and Salvi, 2016; Mannan and Al-Ghamdi, 2021). The concentration of pollutants can be several times higher than outdoors (Simoni et al., 2003). In most cases, outside air is healthier than indoor air (Taştan and Gökozan, 2019). One efficient way to lower the concentration of pollutants at home is to open the windows (Haddad et al., 2021). Health agencies recommend opening the windows to allow fresh air in for a short period of time (at least 10 min each day is recommended).

Knowledge about window opening behavior at home remains scarce. Energy performance in buildings remains the main focus of interest (Fabi et al., 2012; Hou et al., 2017; Park and Choi, 2019). Most models seek to predict manual control of windows at home as a function of outdoor and indoor environmental variables (outdoor and indoor temperature, wind speed, level of PM_2_._5_ and PM_10_, indoor CO_2_ level, relative indoor humidity, etc.) (Arethusa et al., 2014). Unlike many health behaviors, the social and psychological antecedents of window control have not been fully explored until now. The present research addresses a gap in the literature about the volitional and non-volitional antecedents of windows opening. We focus here on the willingness to open windows at home. Our aim is twofold. On the one hand, we will explore the social and psychological antecedents of the intention to open the windows at home. Within the framework of the Theory of Planned Behavior (TPB), we expect the intention to open windows to stem from motivational antecedents available in current social cognitive models of behavior prediction. On the other hand, we will assess the effect of the exposure to a recommendation to open the windows on intention to open the windows. The effectiveness of ventilation recommendations remains an unmapped area, as do the psychological antecedents of window opening.

Indoor air pollution is increasingly covered by the scientific literature as a main health issue (Zang and Smith, 2003; Kampa and Castanas, 2008; Pérez-Padilla et al., 2010; González-Martín et al., 2020; Sakhvidi et al., 2022), whether it stems from the work environment (Reijula and Sundman-Digert, 2004), the home and the daily exposure to pollutants (Missia et al., 2010). The health consequences of air pollution indoors cover a wide range of diseases, from immediate irritation of the eyes and respiratory mucosa (Bernstein et al., 2008) to long-term diseases such as lung cancer and cardiovascular problems (Berglund et al., 1992; Corlan et al., 2021). Most previous studies have focused on pollutants and their multivariate origins: the materials used for construction, biological sources, combustion of materials or occupants’ activities and behaviors (Park and Ikeda, 2006; Kampa and Castanas, 2008; Lin et al., 2017). Behaviors may largely contribute to worsening indoor air quality at home (Kim and Paulos, 2010). Whether it be from smoking habits (Repace and Lowrey, 1993; McAuley et al., 2012), the use of air fresheners/cleansers (Nazaroff and Weschler, 2004) or uses of personal care products (Pieri et al., 2013; Johnson et al., 2019). Indoor air behaviors have been identified as being responsible for a much higher level of fine particle pollution than building characteristics (Spilak et al., 2014). Behaviors related to indoor air can also have a beneficial effect (Park and Choi, 2019), such as the choice of furniture (Lee et al., 2006), or changing habits related to smoking behaviors (Thornley et al., 2013). Fresh air circulation with natural ventilation is an efficient way of maintaining good indoor quality (Saito et al., 2004; Kim et al., 2008; Bekö et al., 2011). Ventilation removes pollutant concentration from an interior space and replaces contaminated air with fresh air from outside (Awbi, 2017; Pan et al., 2019). An inverse relationship was also found between lung cancer and good household ventilation (Jin et al., 2014), and is also a low-cost way of improving air quality at home, which is not complex to implement for most people.

Environmental determinants are the only factors influencing window opening behavior studied by the literature (Fabi et al., 2012; Belafi et al., 2018), with temperature, CO_2_ concentrations, wind speed but also odors (Fabi et al., 2012). Fabi et al. (2012) carried out a review of the factors influencing occupant ventilation behaviors. Different categories were identified, the physical environment, contextual drivers, psychological drivers, and the physiological and social environment. Besides, the climate conditions have a decisive impact on natural ventilation (Escandón et al., 2019). In very hot climate, the ventilation is possible only in a precise moment of the day (night-time) because of extremely hot temperatures. This leads to an opening of windows motivated mainly by environmental factors.

The social dimension needs to be further explored. Fabi et al. (2012) are among the few to integrate a psychological dimension into the drivers of ventilation. However, no study has investigated the psychological determinants of ventilation as the main focus of interest. This exploration could also be used to clarify the effect of environmental cues on behavior when measured with psychological variables.

Environmental cues are susceptible to guide window opening behaviors, especially when they are related to comfort, occupants’ comfort plays an important part in window opening patterns (Mircea et al., 2014; Yao and Zhao, 2017). Odors seriously influence human comfort even if habituation from a prolonged exposure to a compound may decrease the feeling of discomfort (Zhu and Li, 2017). Subjective indoor air quality may also be inferred via cues that are detected by humans (bodily odor, kitchen odors, cleaning agents, etc.), even if many pollutants, such as carbon monoxide, have no detectable odor (Wolkoff, 2018). The absence of odors is not an indication that the place is healthy, and some pleasant odors (e.g., fragrances) may be associated with pollutants (Steinemann and Goodman, 2019; Steinemann et al., 2020; Goodman et al., 2021). Odors may contribute to the opening of windows as cues that are perceived by our sensory abilities as a means of detecting pathogens. Due to the individual’s odor sensitivity (Smeets et al., 2008), it is an interesting variable that may contribute change the willingness to ventilate at home.

Window opening is a repeated behavior that occurs in a predictable pattern of frequency, time of day and places. More window opening is observed in the early morning than at midday (Park and Choi, 2019). This predictable pattern shows potential for the calibration of a TPB application addressed to a behavior with temporal and situational specificities (Ajzen, 1991, 2002).

Considering the lack of research on the social and psychological antecedents of ventilation behavior, the guidelines of the Theory of Planned Behavior (TPB; Ajzen, 1991) will help to organize knowledge. The TPB has been extensively applied to health and environmental issues (Godin and Kok, 1996; De Leeuw et al., 2015), and provides strong predictions of health intention and behavior especially among behaviors that are repeated daily such as handwashing, physical activity and diet (McEachan et al., 2011; Liddelow et al., 2021). Closer to our purpose, Shi et al. (2017) were interested in inhabitants’ behaviors relating to the reduction of targeted pollutants at home. Controlling households’ behaviors relative to PM_2_._5_ greatly helped to reduce pollution. The TPB model explained 56% of the variance of target behaviors. All three antecedents for behavioral intention were predictors for PM_2_._5_ reduction intention. No studies have explored the application of the TPB to window opening. It is fruitful to apply this model to new health behaviors (Rich et al., 2015), especially when those can be explained in a relevant way by the model’s variables as described below.

### Intention

In current life situations, ventilation may be stimulated by an external cue that prompts opening of the windows (Persily, 2015). Even though such cues trigger a behavior with a low cognitive cost, such behavior remains under the control of the will. Occupants may decide to initiate a new behavior or stop the behavior at any time. The first antecedent to opening the windows is therefore a behavioral intention.

### Attitudes Toward Behavior

The evaluation (positive or negative) associated with any discriminable aspect of the world in which we live (Ajzen and Cote, 2008). Attitudes relative to indoor air and window opening has been little explored. In France, Marchand et al. (2018) did not find shared representations of indoor air as such representations are available for other environmental issues (e.g., flood). However, the interviews revealed positive attitudes toward ventilation as a means of protecting health.

### Subjective Norm

The subjective norm is related to the perceived approval of the behavior by the social environment (Ajzen, 1991; Sniehotta, 2009). As people share homes with other occupants (family, roommates, etc.), they may seek others’ approval to decide to open the window. In some cases, opening may be highly approved by significant others for comfort or health motives (Shi et al., 2020). As an expectation, the subjective norm may result from the observation of the number of people who open the window each day (descriptive norm), or from explicit injunctions that inform about what people ought to do (injunctive norm; Cialdini et al., 2006).

### Perceived Behavioral Control

The perceived behavioral control can be broadly defined as the perceived difficulty or ease of establishing the behavior (Ajzen and Driver, 1992). Compared to other health or pro-environmental behaviors (e.g., smoking cessation, regular physical exercise, effective waste management) that are investigated by the TPB model, the opening of windows is easier to learn, easier to perform and easier to apply in one’s own familiar home environment. Except for disabled people who live in inadequate home. So, the manual control of windows is a highly controllable behavior that has positive consequences in the short (fresh and healthy air) and long term (lifelong health), and some immediate negative consequences (exposure to uncomfortable cold air from outdoors, energy expenditure, etc.). Intentions are more likely to be translated into behavior when the targeted behaviors are easy to achieve (Sheeran et al., 2003; Sheeran and Webb, 2016). Perceived Behavior control may moderate the other constructs of the TPB model. Attitudes may lead to favorable intentions and behaviors to the extent that people have a high level of perceived control of the behavior (La Barbera and Ajzen, 2020). Such a moderation effect of perceived control is expected in relation to the opening of windows. Considering the interaction with the subjective norm, a high level of perceived behavioral control is expected to decrease the impact of the subjective norm. The weight of others’ approval may diminish when a behavior is well under control.

### Habits

According to Orbell and Verplanken (2015), habits are defined as converging upon repetition, automaticity and cued by a stable environmental context. Habits are expected to be a predictor of behavior (De Bruijn, 2010; Chen and Chao, 2011; De Bruijn and Rhodes, 2011) and to moderate the relationship between intentions and behavior. In numerous models, the link between habits and intention remains strong and significant (Honkanen et al., 2005; Liddelow et al., 2021). A similar integration of habits in a TPB framed model was found within health-oriented behavior [habits significantly increased the explained variance (fruit consumption, physical exercising)] (De Bruijn, 2010; De Bruijn and Rhodes, 2011). The application of the habit construct to the repetitive feature of window opening behavior may be especially interesting. Verbruggen et al. (2019) defined self-reported habits about ventilation behavior as an action repeated daily before, after or during certain activities, in the everyday life. Also, the relationship between habits and intention is important for non-reflective behaviors (El-Khatib and Barki, 2012).

The effects of indoor air have led health organizations to communicate and establish evidence-based recommendations about indoor air pollution, in the United States with the Environmental Protection Agency (U.S. Environmental Protection Agency, 2003), in the United Kingdom (UK:100, 2019) and in France (INPES, 2009; ANSES, 2018). Detailed instructions include how to ventilate and the health advantages of ventilation. To our knowledge, no research has measured the impact of campaigns or recommendations about ventilation on window opening intentions at home. Considering the small effect size of health messages on behavior (Anker et al., 2016), the probability of a behavioral change following a single recommendation remains low. Nevertheless, other outcomes may benefit from recommendations given by a trustworthy source (e.g., a health agency): the transfer of knowledge, awareness of health importance and a greater willingness to open the windows.

### Overview of Empirical Research and Hypothesis

The extended Theory of Planned Behavior (TPB; Ajzen, 1991, Ajzen and Driver, 1992) provides constructs that are relevant antecedents of ventilation behavior as a goal-directed and a health behavior (Ajzen, 2011). Expectations of ventilation benefits and costs (Attitudes), the subjective ease of opening the windows (Perceived behavioral control), and the perceived social pressure to open or close the windows at home may contribute to the intention to open the windows.

Beyond TPB variables, we will focus on two manipulated contextual variables that may shift intention to open the windows. Firstly, with an exposure to a ventilation health recommendation. Participants will receive information about the health benefit of performing the ventilation behavior. Secondly, with respect to the low level of engagement with indoor air quality, indoor air awareness should increase attention to indoor air as a sensory and physical reality. People who are aware of their own odor sensitivity will agree more with information about the hazards of indoor air pollution and therefore show more intention to open the windows to cope with the health risk (Smeets et al., 2008). In order to control the valence of odors, attention was focused either on positive or negative odors.

We hypothesized that all the predictors of the extended TPB model will play a role in explaining the behavioral intention to ventilate **(H1a)**. We expect that Habits will also contribute **(H1b).** As suggested by La Barbera and Ajzen (2020), we expect that Perceived Behavioral Control may moderate the effect of Attitudes on intention **(H1c)**.

Considering the health recommendation, we expect that it will increase the intention to open windows **(H2a)** and that the effect of the recommendation will be moderated by habits. Contrary to low habit occupants, high habit occupants will not change as a function of a recommendation **(H2b)**. According to odor sensitivity, we expect that people who are aware of their own odor awareness (specially to negative odors) will report intention to open the windows when exposed to a recommendation **(H3a)** and this effect will be moderated by window opening habits **(H3b)**.

Two studies were carried out in France. Study 1 was performed among students. Study 2 replicated the protocol among a larger convenience sample from the Web. The research was conducted in compliance with the Ethical Guidelines of the Psychology Department of the Paris Nanterre University as well as with the National Ethical Standards approved by the University.

## Study 1

The purpose of this study was to weight the psychological determinants of window opening at home. A full TPB model was developed that included the predictors of the intention to open the windows. The effect of a recommendation about ventilation and odor awareness were explored. We organized a student sample. As students spend a lot of time in closed rooms (including educational facilities; Kraus and Nováková, 2019), ventilation is fully recommended among this population. To our knowledge no study suggested a specific behavioral pattern among student samples compared to the general population.

### Methods

#### Participants

Participants (*N* = 230; 15 men; 209 women, 3 others, 3 non-responses) were undergraduates in a French university. Mean age was 19.7 years (*SD* = 3.76; range 18–51). Age was self-reported as an integer in units of years. Seventy-two participants were excluded for the regression analysis for failing to complete all the items of the study (*N* = 159; 9 men and 150 women; Mean age was 19.6 years; *SD* = 3.85).

#### Design

The experimental protocol followed a three-step procedure. Firstly, participants completed (or not) a questionnaire about their abilities to detect positive or negative odors in their environment. The awareness of odor sensitivity was manipulated by a positive (six items) vs. negative (six items) sample of items of the *Odor Awareness Scale* (OAS; Smeets et al., 2008). The goal of the OAS, is to measure sensitivity to positive and negative odors. We used items of this scale not for their primary purpose but as an independent manipulated variable. According to the mere question effect (Godin et al., 2011), questions are by themselves able to change mindset and even behavior in interventions to implement behavior change. Making salient personal sensitivity to detecting odors focuses attention on air as a sensory and physical reality and the valence of air quality. In order to distinguish odor awareness and change in affect, we introduce a self-reported affect measure to control the effect of the awareness of odor sensitivity measure. The induction of a measure relating to positive or negative odors can parasitically induce positive or negative emotions. The purpose of the affect measure is to ensure confounding between a measure of sensitivity to odors and emotions. In the same vein, we introduced an assessment of current indoor air quality to assess the effect of odor awareness manipulation.

Secondly, participants were exposed (or not, presence vs. absence) to a written health recommendation about indoor air and protective behaviors including manual ventilation of their home (10 min/day). The recommendation message (238 words) was delivered by the National Institute for Health Prevention and Education (INPES, 2009 France). The first part of the message explained that dangerous pollutants may be found in the home. The message ended with the following recommendation *“Every day, winter and summer, ventilation for 10 min renews the air in the dwelling and improves the quality of the indoor air.”*

Thirdly, all participants completed a questionnaire about their intention to open their windows at home. The questionnaire measured variables from the extended TPB model. The last questions were about current affect, assessment of current indoor air quality, socio-demographic variables and living conditions. Four treatments (Table 1) were designed as a function of two manipulated variables: Odor awareness and Ventilation Recommendation.

**TABLE 1:** The four treatments design.

Treatment	Factor			
	Study 1	Study 2	Odor awareness	Ventilation recommendation
1	*N* = 58	*N* = 91	Positive	Yes
2	*N* = 53	*N* = 89	Negative	Yes
3	*N* = 49	*N* = 81	No	Yes
4	*N* = 70	*N* = 89	No	No

#### Instruments

##### Habits

The Habits of airing the home measure was based on five items of the 12 items of the *Self Report Habit Index* (SRHI; Verplanken and Orbell, 2003) (e.g., “I *regularly air for 10 min a day*”). The response scale goes from one (Not at all) to five (Very much).

##### Theory of Planned Behavior Constructs

All constructs of the TPB model were developed following the original methodology (Ajzen, 2002). Behavioral Intention (INT) was measured with five items (e.g., “*I intend to open my windows for 10 min every day next week”*). We created eight items to measure Behavioral Attitude (ATT) about ventilation behavior (e.g., *“I think opening the windows for 10 min every day next week would be good for my health”).* Subjective Norm (SN) was measured with six items (e.g., *“Most people I care about think I should open the windows for 10 min every day next week”)* and Perceived Behavioral Control (PBC) with six items (e.g., “*It’s going to be hard for me to open the windows for 10 min every day next week”*). Response modalities for all TPB constructs go from one (Strongly Disagree) to five (Totally Agree).

##### Self-Reported Affect

Current affective state was measured with items selected from the Implicit Positive and Negative Affect Test (IPANAT; Quirin et al., 2009). Contrary to the original scale, we used direct self-reported basic positive and negative emotions. The six-item scale was designed with three positive and three negative emotional words like “*inhibited*,” “*helpless*,” “*happy*.” Modalities of response range from one (Not at all) to five (Strongly) to assess the closeness of participants’ feelings to each emotion displayed.

##### Assessment of Current Ambient Indoor Air

We created a four-item measure of indoor air to assess the indoor air in the place where the participant was currently located (or the last indoor place they were in, for those who might be outdoors during the survey; e.g., *“How do you perceive the air in the room where you are?”*). Response modalities range from one (Very Bad) to five (Very Good).

##### Odor Awareness (Manipulated Between-Subjects Variable)

The manipulation regarding self-assessment of one’s own sensitivity to odors was operationalized by a shortened version of the *Odor Awareness Scale* (OAS; Smeets et al., 2008). We selected six items with either a positive valence (e.g., *Do you feel happy or content when you smell a pleasant odor in the air)* or a negative valence (e.g., *Do you notice the smell of people’s breath or perspiration?*). Response modalities for all items are Never (One); Rarely (Two); Sometimes (Three); Often (Four); Always (Five).

#### Procedure

The study was conducted online from December 2018 to February 2019 (i.e., before the COVID-19 pandemic) using Qualtrics software (Qualtrics, Provo, UT, United States). Informed consent has been asked. Participants were compensated with a partial course credit relative to their first-year curriculum.

### Results

All of the statistics were conducted with The Jamovi Project (2021), exception made for power analysis which was performed on the G^∗^power software.

#### Preliminary Analysis

Descriptive statistics are available in Supplementary Appendix 1. Reliability coefficients of the scales were based on McDonald’s ω (Dunn et al., 2014). Zero-order correlations among all the constructs are available as a Supplementary Material.

The number of participants was calibrated according to the expected effect of a message on change in intentions (Keller and Lehmann, 2008; Anker et al., 2016). A power analysis (G^∗^Power Software, Faul et al., 2009) assumed a small effect size of *d* = 0.25 for the ANOVA with one between-subject factor with four treatments, indicating that a total of *N* = 232 participants were required to have a 90% power of detecting a significant effect at a *p* value of 0.05.

#### Effect of Treatments (Recommendation and Odor Awareness)

A one-way ANOVA assessed the effect of the full design (i.e., four-treatment combination of odor awareness and ventilation recommendation; Table 1) on intention to open the windows (Table 1). The treatment yielded a significant increase of intention, [*F*(3,219) = 3.62, *p* = 0.014, η*^2^* = 0.047]. *Post hoc* Tukey’s indicated that compared with the control group (no recommendation; *M* = 3.87, *SD* = 1.03), recommendation only treatment induced greater Intention to open the windows (*M* = 4.40, *SD* = 0.77; *p* = 0.026; Cohen’s *d* = −0.58). No difference with the control group was found when the ventilation recommendation was previously associated with a positive or negative odor sensibility self-report (respectively, *M* = 4.40, *SD* = 0.77; *M* = 4.02, *SD* = 0.94; and *M* = 4.22, *SD* = 0.80). Considering Affect as a dependent variable, positive (*M* = 2.98) or negative affect (*M* = 2.03) did not change as a function of treatments. Treatments, did not predict current indoor air quality; (*M* = 3.11), *F*(1,190) = 6.22, *p* = 0.014, η*^2^* = 0.031.

#### Antecedents of Behavioral Intention: Hierarchical Regression Analysis

Considering the prediction of the intention to open the windows at home, the aim was to explore the contribution of the extended TPB variables and the manipulated ones (ventilation recommendation and awareness of odors). Exposure to recommendation and odor awareness were coded as dummy variables. The valence of odor awareness was confounded at this step. The recommendation and odor awareness had no effect on the TPB predictors.

A three-step hierarchical linear regression was carried out (Supplementary Appendix 4). Firstly (Model 1), we introduced all the extended TPB model predictors (*i.e., Habits*, *Attitude*, *Subjective Norms*, and *Perceived Behavioral Control)*. Age and gender were added to the model for exploratory purposes. This model explained a large part of variance (*R*^2^ = 0.59, *p* < 0.001). All predictors contributed significantly to explaining the intention to open the windows. The first predictor was *Habits* (*M* = 3.40; β = 0.350, *p* < 0.001). The more participants reported ventilation habits, the more they intended to engage in future ventilation behaviors. Attitudes toward behavior were largely positive (*M* = 4.33, β = 0.244, *p* < 0.001). The analysis revealed a significant effect of Perceived Behavioral Control (*M* = 4.18; β = 0.233, *p* < 0.001). Finally, Subjective Norms (*M* = 3.53) relative to ventilation reached a smaller but significant level (β = 0.197, *p* = 0.001). Age (β = 0.042, *p* = 0.425) had no significant effect on intention. Regarding a very large asymmetry in the sample, no difference was observed due to the gender of participants (β = 0.091, *p* = 0.082).

Following La Barbera and Ajzen (2020), the interaction between Attitude and Perceived Behavioral Control, and Subjective Norm and Perceived Behavioral Control were subsequently added to the model (Model 2). The explained variance slightly increased (*R*^2^ = 0.60) but the increase was above the significance threshold, [*F*(2,150) = 2.15, *p* = 0.120]. Habits and Attitudes and Perceived Behavioral Control remained the main predictors of the intention to open the windows. As illustrated in Supplementary Appendix 4, the interaction between Attitude and Perceived Behavioral Control contributed significantly to the intention to open the windows (β = −0.087, *p* = 0.041). When participants held a higher level of positive attitude toward opening the windows (+1 *SD*), Perceived Behavioral Control was not significantly related to behavioral intention (simple effect, *p* = 0.208). Conversely, among participants with a low level of Attitude (−1 *SD*), Perceived Behavioral Control was significantly and linearly related to the intention to open the windows (simple effect, *p* < 0.01). Interaction between the Subjective Norm and Perceived Behavioral Control yielded a non-significant effect (β = 0.027, *p* = 0.616), but the introduction of this interaction in the model led to the weight of the Subjective Norm as main effect being canceled out.

**FIGURE 1:**
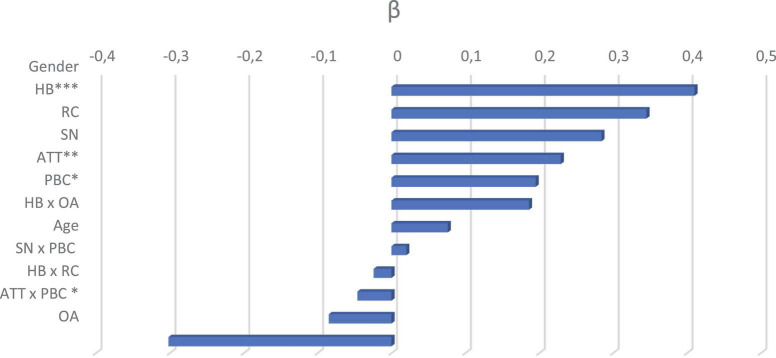
Beta coefficient for the third model in Study 1. ATT, Attitude; SN, Subjective Norm; PBC, Perceived Behavioral Control; HB, Habits; OA, Odor Awareness; RC, Recommendation; ^∗^*p* < 0.05, ^∗∗^*p* < 0.01, ^∗∗∗^*p* < 0.001.

Introduction of Ventilation Recommendation, Odor Awareness (dummy variable) and their interaction with habits (Model 3) increased explained variance (*R*^2^ = 0.60, *p* < 0.001) but did not improve the model significantly, *F*(4,146) = 1.36, *p* = 0.250. Odor Awareness did not contribute significantly to changing intention to open the windows (β = −0.302, *p* = 0.185). The interaction between Odor awareness and habits did not reach the significance threshold. No significant effects were found concerning Exposure to a Ventilation Recommendation and the interaction between Ventilation Recommendation and Habits. As in Model 1 and 2, the main effects of Habits, Attitudes, Perceived Behavioral Control and interaction between Perceived Behavioral Control and Attitudes remained significant. As in Model 2, Subjective Norm and the interaction between Subjective Norm and Perceived Behavioral Control did not contribute significantly to the Model. Gender was above the significance threshold (*p* = 0.07). Age was not significant. The full beta coefficient contributions for this final model, are illustrated in Figure 1.

### Discussion for Study 1

The extended TPB model provided a first framework to predict the intention to open the windows. Habits, Attitude and Perceived Behavioral Control were all positively related to the behavioral intention. As predicted by La Barbera and Ajzen (2020), the interaction between Perceived Behavioral Control and the Subjective Norm canceled out the contribution of the Subjective Norm. At odds with our hypothesis (**H1b**) a significant negative interaction between Perceived Behavioral Control and Attitude showed that Perceived Behavioral Control increased the intention to open the windows only for participants with a low level of Attitude toward behavior. This result may be explained by the ease of manual control of the window as a day-to-day behavior. When people had a very positive Attitude, they did not need to rely on a high level of Perceived Behavioral Control to increase their willingness to perform their behavior. In health domains, the majority of TPB behavior change studies were about behaviors that need a high level of Perceived Personal Control (e.g., physical activity). The recommendation message did not contribute to the extended TPB model. A recommendation message increased, owing to a small effect size, the intention to open the windows but only when the recommendation was not associated with a manipulation of odor awareness. Questions about one’s own sense of odor sensitivity decreased the intention to open the windows and canceled out the effect of the ventilation recommendation (Study 1). A first explanation is that an awareness of one’s own sense of odor sensitivity decreases the perceived vulnerability toward air quality as people are able to detect odors. People often open windows to increase the sensory quality of indoor air (Spentzou et al., 2018). A reminder of their ability to perceive positive or negative odors may lead to feeling less vulnerable toward the risk associated with air pollution, and therefore decrease the need to ventilate.

## Study 2

Using the same design, Study 1 was based on a larger population sample. Two dependent variables were added for exploratory purpose to gain insight about the moderating effect of odor awareness on the recommendation effect on the intention to open the windows (Empowerment and Vulnerability).

### Methods

The four experimental treatments were replicated with the same recommendation message. Addressed to the general population, the questionnaire was shortened with psychometrics analysis, regarding the sample characteristics, to avoid losing too many participants during the online survey.

#### Participants

All the 350 participants (68 men; 272 women, 9 non-responses) were recruited on social media following a Facebook advertisement. Participants were between 18 and 83 years old (*M* = 39.7; *SD* = 14.6; *Md* = 23). Twelve participants were excluded from the regression analysis for failing to fill in all the items of the study (*N* = 338 for the full-regression sample, 68 men; 270 women, between 18 and 83 years old *M* = 39.7; *SD* = 14.5; *Md* = 23). As measured in Study 1, Age was measured as a self-reported integer in units of years. Participants did not receive monetary or non-monetary incentives.

#### Design

The design of this second study is similar to the first study. See Table 1 for a complete illustration of the conditions and their specific characteristics.

#### Instruments

##### Odor Awareness (Manipulated Variable)

The odor sensibility measurement (Smeets et al., 2008) was shortened to three items for the positive items and four for the negative items.

##### Habits

The habits measure for Study 2 was derived from the SRHI scale used in Study 1 (Verplanken and Orbell, 2003). We used a reduced version in three items.

##### Theory of Planned Behavior Constructs

With the same procedure used in Study 1, Attitude (three items), Subjective Norm (three items) and Perceived Behavioral Control were reduced. The five items on Intention to open windows were replicated.

##### Self-Reported Affects

The current affective state was measured with the same adaptation of the Implicit Positive and Negative Affect Test (IPANAT; Quirin et al., 2009) used in Study 1.

##### Assessment of Current Ambient Indoor Air

Participants assess the quality of current indoor air with three items (e.g., “*How do you perceive the air in the room where you are?”*).

##### Empowerment

The items were selected from the Diabetes Empowerment Scale-Short Form (DES- SF; Anderson et al., 2003; Park and Park, 2013). For our purpose, the measure was adapted for indoor air and reduced to three items (e.g., “*I can find ways to reduce indoor air pollution in my home with my knowledge”).*

##### Vulnerability

The three-item measure of vulnerability was inspired by Martens et al. (2017) (e.g., “In *your home, do you think you are exposed to indoor air pollution?*”).

#### Procedure

This experiment was conducted online from November 2019 to January 2020 (i.e., before the COVID-19 pandemic). Informed consented was asked at the beginning of the experiment. Demographic information was recorded at the end of the questionnaire (see Supplementary Appendix 3). Participants were randomly assigned to one of the four experimental treatments. All of the items used a 5-point Likert scale (1–5) where the negative end was 1 and the positive one 5.

### Results

As in Study 1, statistical analyses were conducted on The Jamovi Project (2021).

#### Preliminary Analysis

Similar to Study 1, reliability coefficients of the scales (based on McDonald’s ω; Dunn et al., 2014) are available in Supplementary Appendix 1 and zero-order correlations for the different constructs are available as a Supplementary Material.

#### Effect of Treatments (Recommendation and Odor Awareness)

An ANCOVA was applied to the full effect of treatments on intention and interactions with habits as a covariate. No significant effect was observed nor an interaction between Treatments and Habits. Considering the vulnerability toward the risk of poor indoor air quality, no main effect of treatments was observed, [*F*(3,346) = 0.80, *p* = 0.494] or Habits. Considering the empowerment concerning the risk of poor indoor air quality, no main effect of treatments was observed [*F*(3,346) = 1.82, *p* = 0.142]. No interaction was found between Habits and Treatments. Treatments did not predict the assessment of current indoor air quality; *F*(3,346) = 0.04, *p* = 0.990, η*^2^* = 0.00.

#### Antecedents of Behavioral Intention: Regression Analysis of the Whole Design

A three-step hierarchical linear regression was carried out (see the full detail on Supplementary Appendix 5). First, we introduced the entire extended TPB model as predictors, with age and gender (Model 1). This model explained a large part of variance (*Adj. R^2^* = 0.58, *p* < 0.001). Three predictors contributed significantly to explaining the intention to open the windows. The greater contribution was given by Attitude (*M* = 4.40; β = 0.397, *p* < 0.001), followed by Habits: (*M* = 3.71; β = 0.351, *p* < 0.001) and Perceived Behavioral Control (*M* = 4.09; β = 0.255, *p* < 0.001). No effect was found for Subjective Norm (*M* = 3.40; β = 0.018, *p* = 0.641). Participants’ age (β = −0.06, *p* = 0.096) showed a small non-significant negative trend. Gender (β = 0.062, *p* = 0.484) yielded no effect.

The second step (Model 2; *R*^2^ = 0.59, *p* < 0.001) added interactions between Attitude and Perceived Behavioral Control, and between Subjective Norm and Perceived Behavioral Control. The model increased explained variance compared to Model 1, [*F*(2,329) = 3.89, *p* = 0.021]. The interaction between Attitude and Perceived Behavioral showed a significant negative effect (β = −0.088, *p* = 0.017). Perceived Behavioral Control increased the intention to open the windows more among participants with a low level of behavioral Attitude (−1 *SD*) than a high level of Attitude (+1 *SD*). The interaction between Subjective Norm and Perceived Behavioral Control revealed no effect (β = −0.048, *p* = 0.125).

**FIGURE 2:**
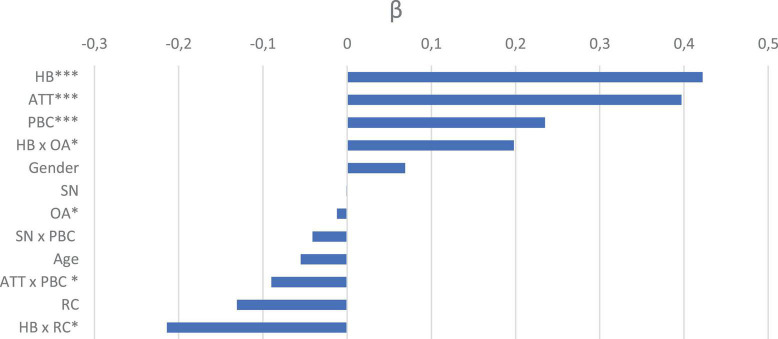
Beta coefficient for the third model in Study 2. ATT, Attitude; SN, Subjective Norm; PBC, Perceived Behavioral Control; HB, Habits; OA, Odor Awareness; RC, Recommendation; ^∗^*p* < 0.05, ^∗∗∗^*p* < 0.001.

The introduction of Ventilation Recommendation and Odor Awareness (as dummy variables) and their interaction with habits (Model 3) did not significantly increase explained variance compared to Model 1, *F*(4,325) = 3.89, *p* = 0.071 (Model 3; *R*^2^ = 0.61, *p* < 0.001). The contribution of Odor Awareness was small but significant (β = −0.012, *p* ≤ 0.031). The interaction of Odor Awareness and Habits (β = 0.198, *p* = 0.021) showed that Odor Awareness decreased intention to open the windows only among low habit participants (−1 *SD*) and not among high habit participants (+1 *SD*). The effect of habits on intention to open the windows was greater when participants were aware of their own odor sensitivity compared with people who were not aware of their odor sensitivity. The exposure to a Ventilation recommendation did not change the intention to open the windows (β = −0.013, *p* = 0.104). The interaction between Ventilation recommendation and Habits (β = −0.214, *p* = 0.032) showed that participants who rated with a high habit for opening the windows (+1 *SD*) decreased their intention after exposure to a recommendation. No effect of the recommendation was observed for participants with a middle or low habit level (Mean or −1 *SD*). Considering the interaction between Attitude and Perceived Behavioral Control (β = −0.097, *p* = 0.005), the same pattern was observed as in Model 1 and 2. Perceived Behavioral Control increased the intention to ventilate but only among participants with a low level of Attitude (β = −0.093, *p* = 0.011). The full beta coefficient contributions for the final model (Model 3), are available in Figure 2.

### Discussion for Study 2

Among a larger convenience sample, the extended TPB model contributed to predicting intention to open windows. The hierarchy of determinants was strong with Attitude and Habits as the main predictors, and Perceived Behavioral Control as secondary predictors. Subjective Norm did not contribute. Interestingly, the negative interaction between Attitude and Perceived Behavioral Control was retrieved. Perceived Behavioral Control increased the willingness to open the windows only among participants who shared a low level of positive Attitude toward ventilation at home. A high level of positive attitude did not need Perceived Control to increase willingness to open the windows. In a larger study, age and gender do not contribute as currently in the TPB model. Unlike in Study 1, we did not find a main effect of the ventilation recommendation or odor awareness. Odor awareness contributed to decrease perceived vulnerability.

## Overall Discussion

Considering the gap in the literature about ventilation as a volitional and non-volitional behavior, our first aim was to identify the psychological determinants of willingness to open windows at home. In the two studies, the extended TPB model has provided significant predictors of intention to open the windows at home, which explained a large amount of variance with around sixty percent of explained variance similar to other health domains (McEachan et al., 2011). In the two convenience samples (students vs. the general population), Habits and Attitude toward behavior were the main predictors. The benefit of good indoor air quality was acknowledged by participants with greater habits compared with participants with low ones. Daily intention to opening the windows may stem both from deliberate intention and from less deliberate processes (habits) that are triggered by external cues (time, place, temperature, etc.) in a stable home environment. This result is consistent with studies showing that habits were found to explain differences in intention, indicating that forming intention does not necessarily have to be reasoned. When a strong habit is present, the expression of an intention might be guided by the salience of past behavior (Honkanen et al., 2005).

After controlling for the interaction with Perceived Behavior Control, social approval did not guide willingness to open the windows in our two samples. The TPB literature about health and pro-environmental behavior predicted a positive interaction between Attitudes and Perceived Behavioral Control, because in behavioral change studies Attitudes need a high level of Perceived Behavioral Control to be translated into behavior (La Barbera and Ajzen, 2020). In our two samples, we found a significant interaction between Perceived Behavioral Control and Attitude toward the behavior. Perceived Behavioral Control increased willingness to open the windows only among people with a low level of Attitude toward the behavior. The specificity of the target behavior must be stressed. The repeated manual control of the window is less challenging than difficult behaviors that have been observed in other health and pro-environmental issues (e.g., regular physical exercise, effective waste management). Positive expectations about the benefits of opening windows do not require a high level of perceived control to motivate window opening.

The second aim of our study was to measure the impact of a recommendation from a public health source on intention. Except for a small effect with our student sample, a single recommendation did not improve the intention to open the windows. Beyond a generic recommendation, effective messages need to be targeted at specific people and places (Petty et al., 2009). We also expected that the lack of awareness of indoor air quality might be improved by a focus about one’s own odor sensitivity. Our hypothesis was firmly rejected. In the first study, the persuasion effect of the ventilation recommendation decreased with odor awareness. In the second study, odor awareness decreased intention to open windows. As expected, the assessment of one’s own ability to detect odor decreased perceived vulnerability to poor indoor air quality.

### Limitations

Student participants (Study 1) and Web-based volunteers (Study 2) may not allow a generalization to wider populations and wider areas. That is not to say that students are not a vulnerable population at home considering indoor air risks (Lai et al., 2018). A larger and representative sample is needed especially with regard to housing conditions and vulnerabilities associated with knowledge, low income, men and women repartition (sample for both Study 1 and Study 2 are constituted with a majority of women) and education levels. Specifically for Study 1, a larger sample size would have been required due to missing responses on the questionnaires used in the hierarchical regression, so these analyses were conducted on a smaller sample than what is recommended by the power analyses, regarding the effect of a recommendation. Nevertheless, the effect size of the prediction of intention by the TPB model is greater (Sheeran and Webb, 2016). Another indication that could be addressed for a larger sample, is taking account for participants local climate conditions. In the framework of our study, our French sample evolves in a temperate climate. The specific characteristics related to climate differences for people living in area with drastic climate differences were not mobilized into the framework of our study. The extension of this research on a large sample allowing the consideration of the climate of individuals on the behavioral determinants of ventilation could be interesting, since these characteristics have been identified to have an effect on behavior (Wei et al., 2017; Escandón et al., 2019).

Another point of improvement relates to the odor awareness manipulation. In Study 2, the strength of our manipulation of odor awareness was plausibly too weak compared to the first study. The focal message (INPES, 2009) was not targeted toward a specific population with a careful consideration of time, context or motivational background of behavior (Keller and Lehmann, 2008). Finally, we did not address the gap between intention and behavior (Sheeran and Webb, 2016). Nevertheless, the specificity of the behavior (to open the windows for 10 min), the global agreement with target behavior and the fact that people have direct experience with it (Glassman and Albarracin, 2006) may narrow the gap with behavior. Regarding the gap between intention and behavior, it seems difficult to provide general guidance on predictors of intention and its power to predict behavior, particularly in the context of meta-analyses concerned with an overall effect of intention on behavior (Armitage and Conner, 2001; Webb and Sheeran, 2006). The weight of determinants in the prediction of intention and behavior varies depending on the application domain (McEachan et al., 2011). Factors related to the generalized prediction of intention and behavior, the magnitude of whose effects vary considerably across behaviors (Rich et al., 2015). The psychosocial antecedents of the window opening behavior at home has not been explored before our research. Further research with a test of the relationship between intention and behavior would fulfill another main gap in the literature. Interestingly, health recommendations did not change intention beyond a student sample. To further illustrates the link between the intention and the behavior, quantitative measures of the behavior are available. Sensors provide an objective measure of window opening and closing (Guerra-Santin et al., 2016).

## Conclusion

Our findings already have practical implications. Considering the lack of knowledge in the ventilation behavior domain, attitude may be a target for public interventions. The health benefits achieved by daily ventilation at home should be acknowledged to a greater extent in multiple risk interventions (Albarracín et al., 2018). Perceived behavior control may be important for more vulnerable people with a less positive attitude toward the regular opening of windows.

Recommendations from public health authorities belong to a large family of intervention techniques (Michie et al., 2018) that may improve human behavior at home for mitigating the dangers of indoor pollutants. We lack knowledge about how home dwellers expect a ventilation gain through the manual control of windows or the fully automatic control of ventilation. Recently the COVID-19 pandemic has made indoor air quality a critical health issue for health public health agencies. Window opening has been added to prevention behaviors among social distancing or handwashing due to the increase of vulnerability to indoor air pollution with COVID-19 (Lyu et al., 2021). The World Health Organisation [WHO] (2021) has given new air quality guidelines, in order to improve the importance of PM_2.5_ as a major health problem. Increasing knowledge about beneficial indoor air behavior such as opening windows has never been more important.

## Data Availability Statement

The original contributions presented in the study are included in the article/Supplementary Material, further inquiries can be directed to the corresponding author.

## Ethics Statement

Ethical review and approval was not required for the study on human participants in accordance with the local legislation and institutional requirements. Written informed consent was not provided because the instruction of the experiment presented to the participants their rights of total retraction at any moment of the experiment as well as in the exploitation of their data. By continuing the manipulation online, they had to agree to give their consent to participate in the study by answering a Yes/No question.

## Author Contributions

FD contributed to the conception and design of the work, data collection, analysis and interpretation, and drafting of the article. BB contributed to the project supervision, conception and design of the work, and data analysis and interpretation. DM contributed to the project management. TM contributed to the project supervision, conception and design of the work, data analysis and interpretation, and drafting of the article. All authors contributed to the article and approved the submitted version.

## Conflict of Interest

The authors declare that the research was conducted in the absence of any commercial or financial relationships that could be construed as a potential conflict of interest.

## Publisher’s Note

All claims expressed in this article are solely those of the authors and do not necessarily represent those of their affiliated organizations, or those of the publisher, the editors and the reviewers. Any product that may be evaluated in this article, or claim that may be made by its manufacturer, is not guaranteed or endorsed by the publisher.
