# Supplementary material for: Psychological Antecedents of the Intention to Open the Windows at Home and Exposure to a Ventilation Recommendation
Source: Front Psychol. 2022 May 13;13:872626. doi: 10.3389/fpsyg.2022.872626 (PMC9136397; doi:10.3389/fpsyg.2022.872626)
Supplement: Supplementary file 1 [file Data_Sheet_1.docx]

**Supplementary material**

| **Correlation Matrix for Study 1.** | | | | | | | | | | |
| --- | --- | --- | --- | --- | --- | --- | --- | --- | --- | --- |
|  |  |  |  |  |  |  |  |  |  |  |
|  | **INT** | **ATT** | **SN** | **PBC** | **HB** | **Positive OA** | **Negative OA** | **Positive affects** | **Negative Affects** | **Assessment of current ambiant indoor air** |
| INT | — |  |  |  |  |  |  |  |  |  |
| ATT | 0.621*** | — |  |  |  |  |  |  |  |  |
| SN | 0.509*** | 0.393*** | — |  |  |  |  |  |  |  |
| PBC | 0.568*** | 0.536*** | 0.301*** | — |  |  |  |  |  |  |
| HB | 0.656*** | 0.322*** | 0.448*** | 0.465*** | — |  |  |  |  |  |
| Positive OA | 0.609*** | 0.518*** | 0.549*** | 0.094 | 0.476*** | — |  |  |  |  |
| Negative OA | 0.233 | 0.190 | -0.012 | 0.180 | 0.164 | NaN | — |  |  |  |
| Positive affects | 0.243*** | 0.174* | 0.229*** | 0.118 | 0.119 | 0.306* | -0.110 | — |  |  |
| Negative affects | 0.026 | 0.028 | 0.099 | 0.125 | -0.027 | -0.125 | -0.014 | 0.474*** | — |  |
| Assessment of current ambiant indoor air | 0.115 | 0.071 | 0.198** | 0.096 | 0.195** | 0.059 | -0.031 | 0.096 | 0.080 | — |

*Note.* ATT = Attitude, SN = Subjective Norm, PBC = Perceived Behavioral Control, HB = Habits, OA = Odor Awareness. **p* <.05, ***p* <.01, ****p* < .001.

|  |
| --- |

| **Correlation Matrix for Study 2.** | | | | | | | | | | | | |
| --- | --- | --- | --- | --- | --- | --- | --- | --- | --- | --- | --- | --- |
|  |  |  |  |  |  |  |  |  |  |  |  |  |
|  | **INT** | **ATT** | **SN** | **PBC** | **HB** | **Positive OA** | **Negative OA** | **Positice affects** | **Negative affects** | **Assessment of current ambiant indoor air** | **Vulnerability** | **Empowerment** |
| INT | — |  |  |  |  |  |  |  |  |  |  |  |
| ATT | 0.585*** | — |  |  |  |  |  |  |  |  |  |  |
| SN | 0.279*** | 0.108* | — |  |  |  |  |  |  |  |  |  |
| PBC | 0.562*** | 0.298*** | 0.250*** | — |  |  |  |  |  |  |  |  |
| HB | 0.614*** | 0.306*** | 0.433*** | 0.552*** | — |  |  |  |  |  |  |  |
| Positive OA | 0.285** | 0.077 | 0.174 | 0.122 | 0.311** | — |  |  |  |  |  |  |
| Negative OA | 0.178 | 0.231* | 0.175 | 0.043 | 0.156 | -0.866 | — |  |  |  |  |  |
| Positive affects | 0.148** | 0.065 | 0.119* | 0.090 | 0.164 | -0.003 | -0.080 | — |  |  |  |  |
| Negative affects | -0.070 | -0.142** | 0.003 | -0.100 | -0.075 | 0.131 | 0.070 | -0.378*** | — |  |  |  |
| Assessment of current ambiant indoor air | 0.103 | 0.011 | 0.123* | 0.121* | 0.234*** | 0.018 | 0.073 | 0.319*** | -0.132* | — |  |  |
| Vulnerability | 0.094 | 0.088 | 0.080 | -0.003 | -0.008 | 0.212* | -0.047 | -0.029 | 0.193*** | -0.333*** | — |  |
| Empowerment | 0.166** | 0.160** | 0.082 | 0.253*** | 0.279*** | 0.023 | 0.065 | 0.175*** | -0.185*** | 0.264*** | -0.044 | — |

*Note.* ATT = Attitude, SN = Subjective Norm, PBC = Perceived Behavioral Control, HB = Habits, OA = Odor Awareness. **p* <.05, ***p* <.01, ****p* < .001.
