# Supplementary material for: Psychological Antecedents of the Intention to Open the Windows at Home and Exposure to a Ventilation Recommendation
Source: Front Psychol. 2022 May 13;13:872626. doi: 10.3389/fpsyg.2022.872626 (PMC9136397; doi:10.3389/fpsyg.2022.872626)
Supplement: Supplementary file 2 [file Data_Sheet_2.docx]

**Appendix 1** Descriptive statistics and consistency of the scales

| Variable | Study 1 | | | Study 2 | | |
| --- | --- | --- | --- | --- | --- | --- |
|  | *M* | *SD* | *ω* | *M* | *SD* | *ω* |
|  |  |  |  |  |  |  |
| INT | 4.10 | 0.92 | .93 | 4.26 | 0.88 | .90 |
| ATT | 4.33 | 0.65 | .86 | 4.40 | 0.71 | .71 |
| SN | 3.53 | 0.80 | .79 | 3.40 | 0.80 | .74 |
| PBC | 4.18 | 0.71 | .75 | 4.09 | 0.86 | .69 |
| HB | 3.80 | 1.27 | .93 | 3.71 | 1.25 | .88 |
| Positive OA | 3.75 | 0.86 | .86 | 3.66 | 0.74 | .58 |
| Negative OA | 3.58 | 0.68 | .73 | 3.54 | 0.83 | .61 |
| Positive affects | 2.98 | 1.03 | .85 | 3.24 | 0.87 | .86 |
| Negative affects | 2.03 | 0.99 | .77 | 1.82 | 0.79 | .68 |
| Assessment of current ambient indoor air | 3.11 | 0.70 | .86 | 3.32 | 0.62 | .81 |
| Vulnerability |  | | | 2.88 | 1.08 | .84 |
| Empowerment |  | | | 3.49 | 0.99 | .85 |

*Note.* ATT = Attitude, SN = Subjective Norm, PBC = Perceived Behavioral Control, HB = Habits, OA = Odor Awareness

**Appendix 2.**

*Socio-demographics variable in Study 1*

|  |  |  |  |  |  |  |
| --- | --- | --- | --- | --- | --- | --- |
| **Living conditions** | (Regression sample) |  |  | (Full sample) |  |  |
| Other | 11 | 6.9% |  | 13 |  | 5.7% |
| Parent's house | 128 | 80.5% |  | 185 |  | 80.4% |
| Apartment sharing | 7 | 4.4% |  | 12 |  | 5.2% |
| In a rental | 13 | 8.2% |  | 20 |  | 8.7% |
| **Room Type** |  |  |  |  |  |  |
| Other | 25 | 15.7% |  | 36 |  | 15.7 % |
| Shared with a roommate | 6 | 3.8% |  | 7 |  | 3.0 % |
| Shared with your partner | 10 | 6.3% |  | 19 |  | 8.3 % |
| Single room | 118 | 74.2% |  | 168 |  | 73.0 % |
|  |  |  |  |  |  |  |

**Appendix 3.**

*Socio-demographics variable in Study 2*

| **Levels** | | **Counts** | | **% of Total** | |
| --- | --- | --- | --- | --- | --- |
| **Housing type** |  |  |  |  |  |
| Apartment |  | 220 |  | 62.9% |  |
| Other |  | 7 |  | 2% |  |
| Individual house |  | 123 |  | 35.1% |  |
| **Living conditions** |  |  |  |  |  |
| Other |  | 12 |  | 3.4% |  |
| Parent's house |  | 27 |  | 7.7% |  |
| Apartment share |  | 27 |  | 7.7% |  |
| As a couple |  | 104 |  | 29.7% |  |
| As a family with children |  | 106 |  | 30.3% |  |
| Alone |  | 74 |  | 21.1% |  |
| **Smoking habits** |  |  |  |  |  |
| Yes |  | 262 |  | 74.9% |  |
| No |  | 88 |  | 25.1% |  |
| **Socio-professional category** |  |  |  |  |  |
| Farmer |  | 1 |  | 0.3 % |  |
| Craftsman, merchant, business owner |  | 23 |  | 6.6 % |  |
| Executive, intellectual profession |  | 111 |  | 31.7 % |  |
| Employee |  | 64 |  | 18.3 % |  |
| Jobseeker |  | 18 |  | 5.1 % |  |
| Student |  | 50 |  | 14.3 % |  |
| Worker |  | 3 |  | 0.9 % |  |
| Intermediary profession |  | 39 |  | 11.1 % |  |
| Retired |  | 31 |  | 8.9 % |  |
| Without professional activity |  | 10 |  | 2.9 % |  |

| **Appendix 4**  *Hierarchical regression analysis of the intention to open the windows for Study 1 (N=159)* | | | | | |
| --- | --- | --- | --- | --- | --- |
| Effect | *SE* | *β* | *B* | 95% *CI* | |
|  |  |  |  | *LL* | *UL* |
|  |  |  |  |  |  |
| Model 1  (*Adj R^2^* =.589***) | | | | |  |
| Intercept | *0.048* | 0.000 | *-0.748* | *-1.690* | *0.195* |
| ATT | *0.068* | 0.244*** | *0.357* | *0.166* | *0.547* |
| SN | *0.057* | 0.197** | *0.220* | *0.088* | *0.351* |
| PBC | *0.067* | 0.233*** | *0.297* | *0.124* | *0.469* |
| HB | *0.059* | 0.350*** | *0.239* | *0.154* | *0.324* |
| Age | *0.013* | 0.043 | *0.010* | *-0.014* | *0.034* |
| Gender^a^ | *0.212* | 0.39 | *0.342* | *-0.044* | *0.728* |
| Model 2  (*Adj R^2^* =.595***; *ΔR^2^* =0.011) | | | | |  |
| Intercept | *0.592* | 0.000 | *-3.090* | *-6.787* | *0.607* |
| ATT | *0.071* | 0.194*** | *1.065* | *0.359* | *1.771* |
| SN | *0.073* | 0.228 | *0.070* | *-0.662* | *0.802* |
| PBC | *0.238* | 0.202** | *0.910* | *-0.038* | *1.783* |
| HB | *0.059* | 0.351*** | *0.240* | *0.156* | *0.324* |
| Age | *0.013* | 0.041 | *0.009* | *-0.015* | *0.033* |
| Gender | *0.210* | 0.379 | *0.330* | *-0.053* | *0.713* |
| ATT x PBC | *0.045* | -0.087* | *-0.185* | *-0.362* | *-0.008* |
| SN x PBC | *0.067* | 0.027 | *0.044* | *-0.128* | *0.215* |
| Model 3  (*Adj* *R^2^* =.599***; *ΔR^2^* =0.0138) | | | | |  |
| Intercept | *0.054* | 0.000 | *-2.953* | *-6.705* | *0.799* |
| ATT | *0.072* | 0.195** | *1.049* | *0.344* | *1.753* |
| SN | *0.059* | 0.229 | *0.089* | *-0.649* | *0.826* |
| PBC | *0.070* | 0.186* | *0.886* | *0.011* | *1.761* |
| HB | *0.060* | 0.345*** | *0.236* | *0.092* | *0.379* |
| Age | *0.013* | 0.020 | *0.004* | *-0.020* | *0.029* |
| Gender | *0.211* | 0.410 | *0.355* | *-0.029* | *0.741* |
| ATT x PBC | *0.045* | -0.085* | *-0.181* | *-0.357* | *-0.004* |
| SN x PBC | *0.054* | -0.024 | *0.039* | *-0.134* | *0.213* |
| OA^b^ | *0.129* | -0.302 | *-0.435* | *-1.081* | *0.211* |
| RC^c^ | *0.142* | 0.284 | *0.352* | *-0.389* | *1.092* |
| HB x OA | *0.125* | 0.076 | *0.052* | *-0.128* | *0.231* |
| HB x RC | *0.140* | -0.046 | *-0.031* | *-0.232* | *0.169* |
| *Note.* ATT = Attitude, SN = Subjective Norm, PBC = Perceived Behavioral Control, HB = Habits, OA = Odor Awareness, RC = Recommendation.  ^a^ 0 = Female, 1 = Male, ^b^ 0 = no Odor Awareness, 1 = Odor Awareness, ^c^ 0 = no Recommendation, 1 = Recommendation; **p* <.05, ***p* < .01, ****p* < .001. | | | | |  |

| \| **Appendix 5** \|  \|  \|  \|  \| \| \| --- \| --- \| --- \| --- \| --- \| --- \| \| *Hierarchical multiple regression: intention to open the windows (N= 338)* \| \| \| \| \| \| \| Effect \| *SE* \| *β* \| *B* \| 95% *CI* \| \| \|  \|  \|  \|  \| *LL* \| *UL* \| | | | | | |
| --- | --- | --- | --- | --- | --- | --- | --- | --- | --- | --- | --- | --- | --- | --- | --- | --- | --- | --- | --- | --- | --- | --- | --- | --- | --- | --- | --- | --- | --- |
|  |  |  |  |  |  |
| **Model 1**  (*Adj R^2^* =.58***) | | | | |  |
| Intercept | 0.034 | 0.000 | 0.177 | -0.330 | 0.683 |
| ATT | 0.036 | 0.397*** | 0.489 | 0.398 | 0.580 |
| SN | 0.038 | 0.008 | 0.008 | -0.077 | 0.093 |
| PBC | 0.048 | 0.255*** | 0.262 | 0.176 | 0.349 |
| HB | 0.045 | 0.359*** | 0.251 | 0.187 | 0.315 |
| Age | 0.002 | -0.06 | -0.004 | -0.008 | 6.45e-4 |
| Gender ^a^ | 0.078 | 0.063 | 0.055 | -0.099 | 0.209 |
| **Model 2**  (*Adj R^2^* =.587***; *ΔR^2^* =0.01*) | | | | |  |
| Intercept | 0.036 | 0.000 | -2.667 | -4.735 | -0.600 |
| ATT | 0.036 | 0.393*** | 0.990 | 0.572 | 1.410 |
| SN | 0.038 | 0.003 | 0.228 | -0.118 | 0.575 |
| PBC | 0.042 | 0.230*** | 0.967 | 0.463 | 1.472 |
| HB | 0.045 | 0.357*** | 0.250 | 0.186 | 0.313 |
| Age | 0.002 | -0.054 | -0.003 | -0.007 | 0.001 |
| Gender | 0.078 | 0.061 | 0.054 | -0.100 | 0.207 |
| ATT x PBC | 0.034 | -0.085* | -0.123 | -0.224 | -0.022 |
| SN x PBC | 0.031 | -0.04 | -0.054 | -0.138 | 0.029 |
| **Model 3**  (*Adj R^2^* =.593***; *ΔR^2^* =0.011) | | | | |  |
| Intercept | 0.035 | 0.000 | -2.886 | -4.953 | -0.817 |
| ATT | 0.036 | 0.397*** | 1.031 | 0.612 | 1.450 |
| SN | 0.038 | -.001 | 0.211 | -0.135 | 0.556 |
| PBC | 0.041 | 0.235*** | 1.000 | 0.497 | 1.503 |
| HB | 0.045 | 0.422*** | 0.295 | 0.189 | 0.401 |
| Age | 0.002 | -0.055 | -0.003 | -0.008 | 9.15e-4 |
| Gender | 0.077 | 0.069 | 0.061 | -0.093 | 0.214 |
| ATT x PBC | 0.034 | -0.090* | -0.131 | -0.232 | -0.030 |
| SN x PBC | 0.031 | -0.041 | -0.053 | -0.136 | 0.030 |
| OA^b^ | 0.084 | -0.012* | -0.504 | -0.961 | -0.047 |
| RC^c^ | 0.097 | -0.131 | 0.443 | -0.092 | 0.978 |
| HB x OA | 0.081 | 0.198* | 0.139 | 0.022 | 0.256 |
| HB x RC | 0.095 | -0.214* | 0.150 | -0.287 | -0.013 |
| *Note.* ATT = Attitude, SN = Subjective Norm, PBC = Perceived Behavioral Control, HB = Habits, OA = Odor Awareness, RC = Recommendation.  ^a^ 0 = Female, 1 = Male, ^b^ 0 = no Odor Awareness, 1 = Odor Awareness, ^b^ 0 = no Recommendation, 1 = Recommendation; **p* <.05, ***p* < .01, ****p* < .001. | | | | |  |
